# Recurrent retrograde intussusception in patient with previous gastric bypass surgery

**DOI:** 10.1093/jscr/rjac414

**Published:** 2022-09-19

**Authors:** Cindy Siaw Lin Wong, Mohamed Ramadan

**Affiliations:** Department of General Surgery, Rotherham General Hospital, Rotherham, South Yorkshire, UK; Department of General Surgery, Rotherham General Hospital, Rotherham, South Yorkshire, UK

**Keywords:** gastric bypass, retrograde intussusception, complications

## Abstract

Retrograde intussusception (RI) is a rare long-term complication of gastric bypass surgery, which usually happens within the first few years after operation. The clinical presentation is vague with overlapping symptoms of bowel obstruction or ischemia. This is a case of a 37-year-old lady who presented with severe abdominal pain. She has background of Roux-en-Y gastric bypass surgery 23 years ago. She underwent emergency laparotomy, bowel resection and Roux-en-Y reconstruction due to finding of ischemic bowel segment. Unfortunately, she had recurrent presentation of RI after 6 months and similar operation was necessitated. She recovered well post-operatively, but there was no definitive cause established for the recurrence. While RI remains a rare diagnosis, clinicians should have high index of suspicion in encountering patients with acute abdomen post gastric bypass surgery. Early imaging is required for the diagnosis and surgical intervention is often warranted.

## INTRODUCTION

Intussusception is a common condition in paediatrics age group. While adult intussusception is rare, it is often pathological due to the presence of lead points such as polyps, Meckel’s diverticulum and neoplasms [1]. In most cases, intussusception occurs along the peristalsis with proximal part of bowel invaginate into distal segment (anterograde). Interestingly, in patients post Roux-en-Y gastric bypass surgery (RYGBP), retrograde intussusception (RI) is more frequently encountered [2]. In this group of patients, the aetiology is largely unknown and mostly occurs without an identified lead point [3]. With the increasing popularity of bariatric surgery, it is imperative for the clinician to be able to recognize this potentially life-threatening condition in patients post-RYGBP.

## CASE PRESENTATION

**Figure 1:**
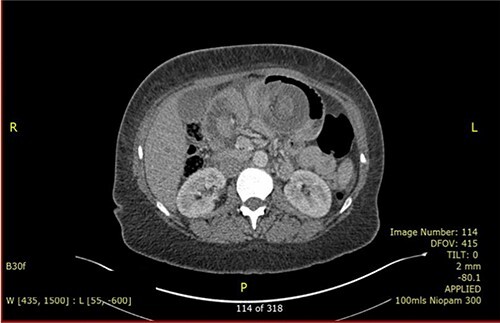
CECT showed the pathognomonic target sign.

A 37-year-old lady presented with 1-day history of sudden onset of severe generalized abdominal pain that was associated with multiple episodes of vomiting. She had occasional similar episodes before, but those were usually milder and were self-resolved. Her past surgical history includes RGYBP surgery 23 years ago and six caesarean sections. She had lost 11 stones of weight in 1 year after the gastric bypass surgery. On physical examination, her abdomen was distended and diffusely tender. Her blood investigation showed low Hb (85 g/dl), raised C-reactive protein (146) and normal white cell count. She had computed tomography (CT) scan (Fig. 1) which concluded intussusception of small bowel with an area of intramural gas highly suspicious of bowel ischaemia. Diagnostic laparoscopy was decided.

**Figure 2:**
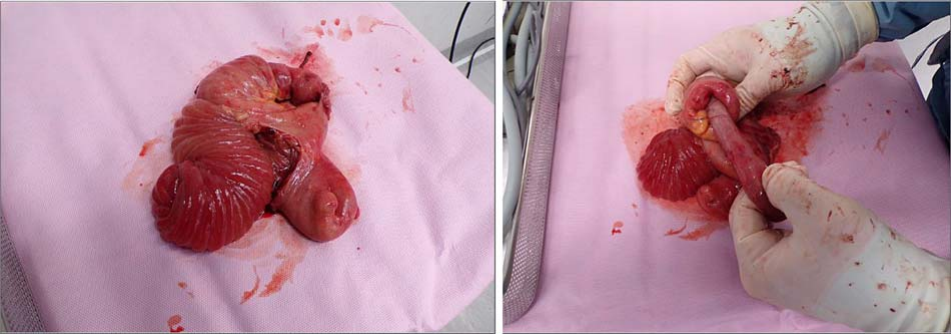
Intraoperative photo showing RI at jejuno-jejunal anastomosis.

Intraoperatively, RI was found at jejuno-jejunal anastomosis involving a long segment of intussusceptum loop going through the jejuno-jejunostomy (Fig. 2), resulting in the obstruction of both alimentary and biliary limbs. Attempt to reduce the intussusception laparoscopically failed hence laparotomy was necessitated. The jejuno-jejunal anastomosis and ~30 cm of non-viable bowel were resected. A new Roux-en-Y anastomosis was created with end-to-end ileo-ileal anastomosis and side-to-side jejuno-ileal anastomosis at the same site. Post-operatively, the patient had a good recovery and was discharged after 1 week.

**Figure 3:**
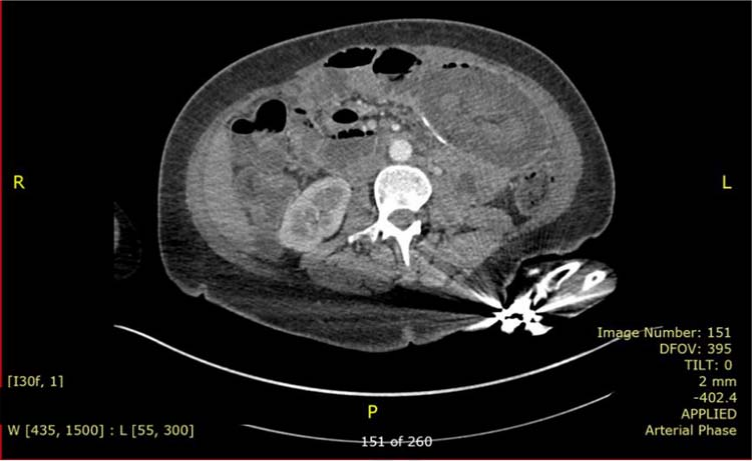
CECT showed the concentric ring sign.

Nevertheless, the patient re-presented 4 months later with similar symptoms. She reported having no issue with bowel after the previous operation. On presentation, her blood results were unremarkable with normal inflammatory markers and lactate. Repeated CT (Fig. 3) again revealed intussusception of small bowel with signs of bowel ischaemia. She was taken to theatre for emergency laparotomy. Intraoperatively, there were dense adhesions with four quadrant purulent peritonitis and a small perforation was found on the anterior staple line of ileo-ileal anastomosis. Distal limb of small bowel had intussuscepted retrograde into the anastomosis, causing obstruction of both thickened proximal limbs. Attempt at the reduction of intussusception was unsuccessful, hence decision was made to proceed with resection and Roux-en-Y reconstruction. Ileo-ileal anastomosis was recreated with stapled side-to-side anastomosis, and roux loop was re-anastomosed just distal to it with double-layered, sutured end-to-side jejunal-ileal anastomosis. Patient made an uneventful recovery and was discharged after a week. Histology of resected small bowel segment in both operations did not show evidence of localized inflammation or neoplasia and the cause of intussusception is not apparent.

## DISCUSSION

RI is an uncommon complication of bariatric surgery with a prevalence of 0.07–0.6% [4]. Most cases occurred within the first few years after RYGBP, and study reported a median time interval of 4 years after the operation [4]. Incidence of recurrence is extremely rare perhaps due to underdiagnosed cases, especially in the case of self-reduced intussusception as the symptoms are non-specific. RI in adults seldom present with classical triad of cramping abdominal pain, bloody diarrhoea and palpable mass as seen in paediatrics case. In most of the case reports, patients presented with non-specific symptoms spanning across a spectrum of severities, more commonly abdominal pain, obstructive symptoms, altered bowel habit or abdominal distension [5]. In this case, the patient presented with complications of intussusception, bowel ischaemia in both episodes.

### Pathophysiology

Multiple hypotheses have been postulated regarding the pathophysiology behind RI. Hocking *et al*. has proposed the theory of motility disorder of Roux limbs [6]. The theory suggested that there is disturbance in pacemaker cells electrical activity as Roux limbs is created, leading to the erratic propagation of electric signals in the joined bowel segments. Another popular hypothesis is that significant weight loss after RYGBP causes elongation of thinning mesentery and increases chances of bowel telescoping due to relative free movement of bowel [7]. On top of that, there are some common lead points which can cause intussusception such as reactive lymph nodes, adhesions, suture or staple line [8]. With the prevalence of cases in female patients [7], it is controversial whether hormonal factors will play a role in the aetiology of RI. In this patient, it is still far from explanation the reason of long-time frame for intussusception to develop after RYGBP.

### Diagnosis and treatment

CT scan is the preferred imaging modality for early identification of intussusception [9]. Characteristic findings are target signs and multiple concentric rings as seen in this case. While CT can diagnose intussusception, there is limitation in identifying its underlying aetiology. Other imaging modalities, such as plain abdominal films or ultrasonography, can be used but is less practical in diagnostic purpose compared to CT scan.

To date, there is no definitive treatment recommended for adult intussusception due to the rarity of cases [10]. Generally, management depends on the severity of patient’s condition and whether there is high suspicion of bowel ischaemia, but surgical exploration is usually warranted. Various surgical options have been adopted, including manual reduction with or without plication and fixation of the mobile efferent limb to adjacent tissues [11]. A case review has suggested that resection and reconstruction regardless of bowel viability has better efficacy in obviating recurrence than manual reduction alone (7.7 vs. 33.3%) [2]. However, in this case, the patient had recurrence after bowel resection and reconstruction of Roux limbs, hence the chances of recurrence remain unpredictable.

## CONCLUSION

RI, although rare, is an important diagnosis to be aware of when encountering patients with acute abdomen with history of gastric bypass surgery. There should be a low threshold for performing CT scan early in such patients as prompt diagnosis is imperative to prevent developing bowel ischaemia. Further management will be guided by the patient’s general condition and any doubt of bowel viability. Patients should be alerted about the possibility of recurrence and should seek early consultation in the presence of symptoms.

## CONFLICT OF INTEREST STATEMENT

The authors declare that there are no competing interests.

## References

[1] Ali BI , et al. Intussusception after roux-en-y gastric bypass in a pregnant patient. J Med Case 2016;7:260–2.

[2] Daellenbach L , Suter M. Jejunojejunal intussusception after Roux-en-Y gastric bypass: a review. Obes Surg 2011;21:253–63. 20949329 10.1007/s11695-010-0298-5

[3] Marinis A , et al. Intussusception of the bowel in adults: a review. World J Gastroenterol 2009;15:407–11. 19152443 10.3748/wjg.15.407 PMC2653360

[4] Simper SC , et al. Retrograde (reverse) jejunal intussusception might not be such a rare problem: a single group’s experience of 23 cases. Surg Obes Relat Dis 2008;4:77–83. 18294922 10.1016/j.soard.2007.12.004

[5] Kumar A , Ogbonda S, Persaud P, Shiwalkar N. Retrograde intussusception after Roux-en-Y gastric bypass. Cureus 2020;12:e8825. 10.7759/cureus.8825 PMC7320641 32607307

[6] Hocking MP , McCoy DM, Vogel SB, Kaude JV, Sninsky CA. Antiperistaltic and isoperistaltic intussusception associated with abnormal motility after Roux-en-Y gastric bypass: a case report. Surgery 1991;110:109–12. 1866683

[7] Singla S , Guenthart BA, May L, Gaughan J, Meilahn JE. Intussusception after laparoscopic gastric bypass surgery: an underrecognized complication. Minim Invasive Surg 2012;2012:464853. 10.1155/2012/464853 PMC3444049 22991661

[8] Zainabadi K , Ramanathan R. Intussusception after laparoscopic Roux-en-Y gastric bypass. Obes Surg 2007;17:1619–23. 18043990 10.1007/s11695-007-9291-z

[9] Gayer G , Zissin R, Apter S, Papa M, Hertz M. Pictorial review: adult intussusception–a CT diagnosis. Br J Radiol 2002;75:185–90. 11893645 10.1259/bjr.75.890.750185

[10] Maghrebi H , Makni A, Rhaiem R, Atri S, Ayadi M, Jrad M, et al. Adult intussusceptions: clinical presentation, diagnosis and therapeutic management. Int J Surg Case Rep 2017;33:163–6. 28327421 10.1016/j.ijscr.2017.02.009 PMC5358816

[11] Varban O , Ardestani A, Azagury D, Lautz DB, Vernon AH, Robinson MK, et al. Resection or reduction? The dilemma of managing retrograde intussusception after Roux-en-Y gastric bypass. Surg Obes Relat Dis 2013;9:725–30. 22738754 10.1016/j.soard.2012.05.004

